# Investigation of the clinical utility of the hypomania checklist 32 (HCL-32) for the screening of bipolar disorders in the non-clinical adult population

**DOI:** 10.1186/s12888-016-0831-8

**Published:** 2016-05-04

**Authors:** Kounseok Lee, Hyeji Oh, Eun-Ho Lee, Joo Hyun Kim, Ji-Hae Kim, Kyung Sue Hong

**Affiliations:** Department of Psychiatry, Sungkyunkwan University School of Medicine, Samsung Medical Center, 50 Irwon-dong, Gangnam-gu, Seoul, 135-710 Republic of Korea; Samsung Biomedical Research Institute, Samsung Medical Center, Seoul, Republic of Korea; Depression Center, Samsung Medical Center, Seoul, Republic of Korea; Present address: Department of Psychiatry, St. Andrew’s Hospital, Icheon, Republic of Korea

**Keywords:** Bipolar disorder, Screening, Hypomania checklist-32

## Abstract

**Background:**

The hypomania checklist-32 (HCL-32) is a widely used questionnaire developed for identifying hypomanic components in patients with a depressive episode. Measuring and screening previous hypomanic symptoms in individuals without any definite history of depressive episode would also be needed for early detection of bipolar disorders (BDs). This study aimed at testing the clinical utility of the HCL-32 for screening of BDs in the non-clinical population.

**Methods:**

Lifetime history of hypomanic symptoms was evaluated by using the HCL-32 in 220 patients with BDs and 313 non-clinical individuals. Sensitivity, specificity, and the area under the curve (AUC) of the Receiver Operating Characteristic (ROC) were evaluated for assessing the discriminatory power of the scale and its two sub-domains in screening BDs.

**Results:**

The mean HCL-32 total score was significantly higher in the Bipolar II disorder group compared to the non-clinical group (*P* < 0.001). Most of the items (10/12) of the irritable/risk-taking factor showed higher positive responses in patient groups. Items of active/elated factor showed mixed results. The HCL-32 total score and the active/elated factor score were not adequate for both BDs and its subgroups with AUC values of less than 0.7. The irritable/risk-taking factor score showed higher discrimination power, i.e. AUC for BDs, Bipolar I disorder, and Bipolar II disorder was 0.71, 0.67, and 0.75, respectively.

**Conclusions:**

The HCL-32 could not adequately distinguish BD patients from the non-clinical adult population. However, the current study identified items of irritable/risk-taking factor of the scale that could be useful in screening BDs in the general population.

## Background

It is well known that assessing previous incidents of hypomania requires special attention and caution in psychiatric practice [1]. Individuals do not always perceive hypomania as pathological, and as such do not spontaneously report it to clinicians [2]. Therefore, the recognition of hypomania is frequently delayed, distorting the accurate identification of diagnosis [3]. Individuals with bipolar disorder (BD) often report that the illness manifested itself early in their life, but accurate diagnosis lagged by many years [4]. Failure to diagnose or misdiagnosis in BD is a significant clinical and public health problem, as it brings about delayed intervention and an unfavorable treatment outcome.

The need to improve the recognition and diagnosis of hypomania has led to the development of self-report questionnaires for bipolar disorder, including the Mood Disorder Questionnaire (MDQ) [5], and the Hypomania Checklist (HCL-32) [6]. The HCL-32 is a self-report questionnaire developed to identify hypomanic components in patients with major depressive episodes [6]. It has been used as a screening instrument for BDs and bipolar spectrum disorders in various psychiatric clinical settings [7]. The HCL-32 showed an adequate discrimination power for distinguishing individuals with BDs from those with major depressive disorders in previous studies [6–9]. Those studies also identified two or three sub-domains of the scale representing separate clinical factors. Compared to the MDQ, the HCL-32 showed higher sensitivity and lower specificity in screening hypomania, having high accuracy for the detection of ‘softer’ BD cases [10]. However, both questionnaires were developed for and have been applied to patients in a depressive episode.

Even though a manic/hypomanic episode is usually preceded by one or more depressive episodes, the polarity of the first episode of illness in BDs is manic (or hypomanic) in at least one third of patients [11, 12]. Untreated hypomania may be associated with marital, financial, legal, occupational and other psychosocial problems [13]. Therefore, screening previous and current hypomanic symptoms in individuals without any clear depressive episode would also be important for early detection and adequate treatment of BDs.

The aim of the current study was to test the clinical utility of the HCL-32 for the screening of BDs in the non-clinical adult population. Considering that bipolar I disorder (BD1) and bipolar II disorder (BD2) might have somewhat discrete clinical natures, and that the development of the HCL-32 was originally more focused on BD2 than BD1 [6], the same analyses were performed separately for these two diagnoses. Additionally, we analyzed both total score and sub-domain scores of the HCL-32.

## Methods

### Participants

We used patients’ data from the HCL-32 included in the comprehensive psychological test that has been applied to outpatients and inpatients of the Samsung Medical Center. Patients had been consecutively referred by their attending psychiatrists for psychological evaluation from March 2006 to September 2010. Among these patients, we selected patients who met the diagnostic criteria of the Diagnostic and Statistical Manual of Mental Disorders, 4^th^ edition (DSM-IV) BD1 and BD2 as the study subjects. Clinical diagnosis was made by the attending psychiatrist of each patient. Additionally an independent diagnosis with a structured clinical interview using the Korean version of the Structured Clinical Interview of DSM-IV [14] or the Korean version of the Mini International Neuropsychiatric Interview [15] was made by a well-trained psychologist. The exclusion criteria were 1) patients whose clinical diagnosis and diagnosis by a structured interview were not consistent, 2) patients in unstable or severe clinical status who could not cooperate with the study procedures, and 3) individuals who were illiterate, suffering from mental retardation, dementia or intellectual impairment. We received written consent from the subjects for the use of their psychological test data from clinical researches of Samsung Medical Center. Finally, data of 112 patients with BD1 and 108 patients with BD2 were included in the analyses. The sample included 145 (65.9 %) inpatients and 75 (34.1 %) outpatients. The patients’ most recent episode was as follows: manic, 65 (12.1 %); hypomanic, 11 (2.1 %); mixed, 16 (3.0 %); depressed, 16 (3.0 %); and unspecified, 4 (0.8 %). Only ten patients were in remission at the time of psychological assessment. Healthy subjects between 18 and 60 years of age were recruited from the local community from February to June in 2012 as a control group for the BD studies of our research group. Detailed descriptions on the control group were also presented in a previous paper from our group [16]. Individuals with any history of psychiatric illness, including any mood episode, mental retardation, substance abuse, or medical illness and medications known to affect moods were excluded [16], and data of 313 individuals were included in the current analysis. Written informed consent was obtained from all participants for the use of their test data in the BD studies of our research group. This study was approved by the Institutional Review Board of the Samsung Medical Center.

### Measures

#### Application of the Hypomania Checklist (HCL-32)

The HCL-32 comprises 32 “yes” or “no” questions on hypomanic symptoms appearing in emotions, behaviors, or thoughts. Participants are requested to remember ‘a period when you were in a “high” state’ and to indicate if specific behaviors, thoughts, or emotions were present in such a state. The total score is the number of items with a “yes” response. Two sub-domains have been consistently identified through previous studies, i.e. active/elated and irritable/risk-taking factors [6, 17–19]. Classification of individuals to the sub-domains that show minor differences among studies was conducted according to the sub-domain structure of the large-scale transcultural study of Angst et al. [17]. We used the validated Korean version of the HCL-32 [20, 21].

#### Analysis

Differences in demographic characteristics between patient groups and the control group were evaluated using the analysis of variance (ANOVA) for continuous variables and the χ^2^ test for dichotomous variables. The area under the curves (AUC) of the receiver operating characteristic (ROC) curves were evaluated to determine the discriminatory power of the HCL-32 and its sub-domains in screening BD. The score with the highest combination of sensitivity and specificity (Youden's index) was determined as the optimal cut-off score [22]. The overall performance was expressed by the area under the ROC curve (AUC), defining performances as follows: excellent (0.90–1.00), good (0.80–0.89), fair (0.70–0.79), poor (0.60–0.69) or very poor (<0.60) [23]. The positive predictive value (PPV) was calculated by dividing the number of true positives by the number of true positives plus false positives. Negative predictive value (NPV) was calculated by dividing the number of true negatives by the number of true negatives plus false negatives.

IBM SPSS 20.0 (IBM corp., Armonk, NY, USA) and R 3.0.2 (R Development Core Team, Vienna, Austria, http://www.R-project.org/) with pROC [24] and qvalue [25] packages was used for statistical analyses. *P* value < 0.05 was considered as statistically significant. Analysis of false discovery rate (FDR) [26] was applied to control for experiment-wise type I errors resulting from multiple comparisons. The FDR procedure controls for the expected proportion of false discoveries (i.e. incorrectly rejected null hypotheses) by providing an adjusted overall q-value, with adjusted p-values for each comparison. The FDR threshold was set to 0.05.

## Results

The demographic and clinical characteristics of the subjects are presented in Table 1. There was no significant difference in age and sex among subject groups. Educational level was significantly higher in the control group compared to patient group (*P* < 0.001).

**Table 1:** Sociodemographic and clinical characteristics of the subjects (*n* = 533)

	Control (*n* = 313)	BD1 (*n* = 112)	BD2 (*n* = 108)	Statistics (F or χ^2^)	*P*-value	Post hoc
Age, years (mean ± SD)	33.5 ± 9.2	33.9 ± 1.3	35.0 ± 1.3	0.0734	0.481	
Sex				3.766	0.15	
Male	122 (39.0 %)	39 (34.8 %)	31 (28.7 %)			
Female	191 (61.0 %)	73 (65.2 %)	77 (71.3 %)			
Education, n (%)				36.162	<0.001	Control > BD1, BD2
College graduate or more	254 (81.1 %)	65 (58.6 %)	61 (56.6 %)			
Hypomania scores (mean ± SD)						
HCL-32 total	14.7 ± 5.4	16.1 ± 6.7	17.6 ± 6.5	10.527	<0.001	BD2 > Control
Active/elated	12.4 ± 4.7	11.9 ± 5.0	13.0 ± 4.9	1.401	0.247	
Irritable/risk-taking	2.2 ± 1.8	3.9 ± 3.0	4.5 ± 2.8	49.280	<0.001	BD2, BD1 > Control

Post hoc: Scheffe’s test for continuous measures, pair-wise chi-square test for categorical measures

*BD1* bipolar I disorder, *BD2* bipolar II disorder, *HCL-32* hypomania checklist 32

Patient groups showed higher HCL-32 total score compared to the control group. However, a statistically significant difference was observed only between the BD2 group and the control group (*P* < 0.001). For the active/elated factor score, there was no significant difference among subject groups (*P* = 0.307). Irritable/risk-taking score was highest in the BD2 group and there was a significant difference among groups (*P* < 0.001, control < BD1 < BD2). The positive response rate of each item is presented in Table 2. Ten of 12 items (83.3 %) of the “irritable/risk-taking” factor showed a higher positive response rate in patient groups compared to the control group. Among the 19 items of active/elated factor, 13 items (68.4 %) showed no group difference, and 3 items revealed a significantly higher positive response rate in the control group compared to BD1.

**Table 2:** Comparison of positive response rate of HCL-32 items among the subjects groups

		Positive response (%)			χ^2^	*P* value		post-hoc
		Control (*n* = 313)	BD1 (*n* = 112)	BD2 (*n* = 108)		normal	FDR	
*Active/elated factor*								
1	need less sleep	53.0	53.6	63.0	3.344	0.188	0.27	
2	more energetic	79.6	75.9	84.3	2.398	0.301	0.42	
3	more self-confident	77.9	78.6	77.8	0.027	0.987	0.97	
4	enjoy my work more	78.6	73.2	77.8	1.378	0.502	0.61	
5	more sociable	70.6	67.9	70.4	0.307	0.858	0.93	
6	want to travel more	59.7	43.8	49.1	9.947	0.007	0.02	Control > BD1
10	physically more active	52.9	48.6	55.1	0.977	0.613	0.69	
11	plan more activities	77.6	64.3	83.3	12.064	0.002	0.01	BD2, Control > BD1
12	have more ideas/creative	65.5	72.3	67.6	1.752	0.417	0.53	
13	less shy	62.3	70.4	70.1	3.552	0.169	0.26	
14	wear more extravagant clothes	37.4	41.4	54.2	9.292	0.01	0.02	BD2 > Control
15	meet more people	61.9	57.7	57.0	1.102	0.576	0.67	
16	more interested in sex	30.8	34.8	45.8	7.945	0.019	0.04	BD2 > Control
18	talk more	64.9	71.4	79.4	8.291	0.016	0.03	BD2 > Control
19	think faster	66.1	65.8	73.1	1.988	0.37	0.49	
20	make more jokes	73.8	60.4	70.4	7.091	0.029	0.05	
22	engage in more new things	77.6	75.9	75.9	0.199	0.905	0.95	
24	do more quickly/easily	68.4	68.5	69.4	0.044	0.978	0.97	
28	mood higher, more optimistic	80.8	67.6	71.3	9.672	0.008	0.02	Control > BD1
*Irritable/risk-taking factor*								
7	drive faster	10.0	21.6	27.9	21.993	<0.001	<0.01	BD1, BD2 > Control
8	spend more	39.3	48.2	61.1	15.796	<0.001	<0.01	BD2 > Control
9	take more risks	9.3	25.9	34.6	41.081	<0.001	<0.01	BD1, BD2 > Control
21	more easily distracted	29.7	40.2	48.6	13.619	0.001	<0.01	BD2 > Control
23	thoughts jump	45.4	60.7	65.4	16.58	<0.001	<0.01	BD1, BD2 > Control
25	more impatient/irritable	14.7	44.1	40.2	50.986	<0.001	<0.01	BD1, BD2 > Control
26	can be exhausting or irritating	5.1	32.4	27.1	61.865	<0.001	<0.01	BD1, BD2 > Control
27	get into more quarrels	2.9	24.3	23.4	55.664	<0.001	<0.01	BD1, BD2 > Control
29	drink more coffee	30.7	31.5	41.1	4.07	0.131	0.21	
30	smoke more cigarettes	5.8	22.7	23.8	34.864	<0.001	<0.01	BD1, BD2 > Control
31	drink more alcohol	23.0	22.5	33.0	4.673	0.097	0.16	
32	take more drugs	0.6	24.1	14.2	65.772	<0.001	<0.01	BD1, BD2 > Control

*BD1* bipolar I disorder, *BD2* bipolar II disorder, *FDR* false discovery rate, *Post hoc* Pair-wise chi-square test

The AUC of the ROC curve and optimal cut-off scores of the HCL-32 and 2 sub-domains for discriminating patients groups from the control group are summarized in Table 3. The AUC range was between 0.51 and 0.75. An AUC of 0.7 or higher was observed only in the irritable/risk-taking factor for discriminating BD and BD2 from the control group. The specificity and sensitivity for screening BD at the optimal cut-off score are also presented in Table 3. The HCL-32 total score showed high specificity (0.82–0.93) and poor sensitivity (0.31–0.36). The irritable/risk-taking factor score had acceptable ranges of specificity (0.62) and sensitivity (0.65–0.74).

**Table 3:** Analyses of the discrimination power of HCL-32 and its sub-domains in screening BDs

	HCL-32 total	Active/elated	Irritable/risk-taking
BD1 vs Control			
AUC (95 % CI)	0.56 (0.51–0.61)	0.52 (0.47–0.57)	0.67 (0.62–0.72)
optimal cut-off score	>19	≥12	>2
PPV (%)	38.87 (28.89–49.60)	29.72 (23.42–36.65)	37.96 (31.07–45.24)
NPV (%)	77.72 (72.22–81.52)	76.53 (70.50–81.86)	83.20 (77.77–87.76)
sensitivity (95 % CI)	0.32 (0.24–0.42)	0.52 (0.42–0.62)	0.65 (0.55–0.74)
specificity (95 % CI)	0.82 (0.77–0.86)	0.56 (0.50–0.62)	0.62 (0.56–0.67)
BD2 vs Control			
AUC	0.64 (0.60–0.69)	0.54 (0.49–0.59)	0.75 (0.70–0.79)
optimal cut-off score	>21	>11	>2
PPV (%)	53.84 (43.54–71.23)	27.20 (22.06–32.84)	37.63 (30.82–44.81)
NPV (%)	81.31 (76.95–85.17)	83.27 (76.12–88.99)	88.50 (83.60–92.36)
sensitivity (95 % CI)	0.31 (0.22–0.40)	0.76 (0.66–0.83)	0.74 (0.64–0.82)
specificity (95 % CI)	0.93 (0.90–0.96)	0.37 (0.32–0.43)	0.62 (0.56–0.67)
BDs vs Control			
AUC	0.60 (0.56–0.65)	0.51 (0.47–0.56)	0.71 (0.67–0.75)
optimal cut-off score	>19	>10	>2
PPV (%)	58.44 (49.66–66.83)	43.31 (38.28–48.46)	56.07 (49.94–62.07)
NPV (%)	64.57 (59.65–69.28)	63.82 (55.64–71.45)	73.99 (68.24–79.19)
sensitivity (95 % CI)	0.36 (0.30–0.43)	0.75 (0.68–0.80)	0.69 (0.62–0.75)
specificity (95 % CI)	0.82 (0.77–0.86)	0.31 (0.26–0.37)	0.62 (0.56–0.67)

Cut-off score with the highest Youden’s Index value

*AUC* the area under the curve of the receiver operating characteristic (ROC) curve, *HCL-32* hypomania checklist 32, *BD1* bipolar I disorder, *BD2* bipolar II disorder, *BDs* bipolar disorders, *PPV* positive predictive value, *NPV* negative predictive value

## Discussion

This study investigated the clinical utility of the HCL-32 for screening BDs in the non-clinical general population. The HCL-32 total score and active/elated factor score did not have adequate screening properties to differentiate patient groups from the control group. However, the irritable/risk-taking factor score with an AUC range of 0.67–0.75 was better able to differentiate between the two groups.

Demographically, female dominance was observed in both the patient group and the control group and had no significant difference among groups. According to a systematic review of prevalence studies, the mean prevalence rate of BDs for males and females was 0.625 % (SD: 0.679) and 0.968 % (SD: 0.945), respectively [27]. Another review of the prevalence of BDs in general primary care samples also reported higher prevalence in females [28]. Female dominance has been observed frequently in clinical studies that recruited consecutive cases of BD patients [29, 30]. Regarding education, the control group was biased to higher educational level, which might affect the positive response rate of their previous mood experiences.

The scores for the patients groups in the current study (16.1 for BD1, and 17.6 for BD2) were similar to those of the initial report of Angst et al. [6] (18.2 for BD1, and 17.3 for BD2) and other previous studies, i.e., 15.4–18.8 for BD1, and 17.3–18.1 for BD2 [9, 21]. The mean HCL-32 total score of the control group in the current study (14.7) was similar to those of previous studies, i.e., 15.8 [31] and 15.7 [19]. However, the HCL-32 total score of the control group was higher than that of depression patients in previous studies, i.e., 7.9–14.5 [6, 8, 9, 32, 33]. As a result, the cut-off scores of the HCL-32 yielding the best combination of sensitivity and specificity for screening BDs in the present study (19–21) were higher than the cut-off score of 14 that has been accepted as optimal for discriminating BDs from MDD in previous studies [6, 8, 32, 34]. In analyzing individual items, high HCL-32 total scores of the control group resulted from a high positive response rate (30.8 % ~ 80.8 %) to items of active/elated factor. This result might indicate that normal persons are more likely to report their experience of positive or high mood state than depressive patients.

The majority of items of irritable/risk-taking factor showed a higher response rate in patient groups in contrast to non-specific high positive response to items of active/elated factor. This phenomenon is indicated by the much better discrimination properties (AUC and sensitivity and specificity at optimal cut-off score) of the risk-taking/irritable factor score compared to the HCL-total score and active/elated factor score. These results are consistent with results of a previous study by Meyer et al. in which German people with probable episodes of hypomania showed a higher score only in the ‘risk-taking/irritable’ factor (not in the ‘active/elated’ factor) compared to the non-hypomanic group [19]. Other studies also indicated that irritable/risk-taking symptoms might have more relevance to bipolarity and related psychopathological conditions [19, 31]. In a study of an adolescent group, ‘irritable/erratic’ factor and ‘disinhibited/stimulation-seeking’ factor of the HCL-32 were significantly associated with behavioral and cognitive problems that are prevalent in BDs, while ‘active/elated’ factor negatively correlated with peer problems [31]. Participants in another adult study cohort reporting irritable/risk-taking hypomania had more depressive symptoms, sleep disturbances, somatic complaints, perceived stress, and lower self-efficacy compared to those reporting active/elated hypomania or no hypomania [35].

We applied the same analyses in the present study to BD and two subgroups (BD1 and BD2). The HCL-32 total and sub-domains scores showed better discrimination ability in BD2 compared to BDs and BD1. Previous manic episodes are much easier to detect than hypomanic episodes in patients with depressive episodes [34]. Therefore, the HCL-32 was developed to focus on the identification of hidden hypomanic components in patients with depressive episodes in order to obtain reliable diagnoses of BD2 and bipolar spectrum disorders [15]. The validity of the HCL-32 in the detection of BD2 and minor BDs in patients with depressive episodes was demonstrated in subsequent studies [6, 34]. The present study shows that most of the items of irritable/risk-taking factor of the HCL-32 could be useful in the screening of hypomanic state of the BD2 in the non-clinical population.

There are certain limitations to be considered in the interpretation of the present results. First, psychiatric assessments using the structured clinical interview were not performed for the control group which could lead to inclusion of un-diagnosed BD patients in the control group. Second, as patients were recruited in a single psychiatric unit, they could not represent BD patients in general in terms of demographic and clinical characteristics. Third, the current mood state of the patients could have yielded a recall bias on their previous hypomanic state assessed in the HCL-32. Last, differences in the education level between the patients and control groups could be an uncontrolled confounding factor.

Within the mentioned limitations, the present study is meaningful as the first study to evaluate the clinical utility of HCL-32 for screening BDs in non-clinical samples. Our findings indicated that even though the HCL-32 total score and active/elated factor score could not adequately screen BDs from the general population, irritable/risk-taking sub-domain could be clinically useful for distinguishing BDs from the control groups. Beyond its quality as a screening instrument, future studies would be required to explore whether the irritable/risk-taking sub-domain of the HCL-32 might also be useful in assessing the severity measures of the illness in the course of BD.

## Conclusions

This study investigated the clinical utility of the HCL-32 for the screening of BDs in the non-clinical general population. The HCL-32 total score and active/elated factor score did not show adequate screening properties for differentiating patient groups from the control group. However, the current results suggest that items of irritable/risk-taking factor could be useful in screening BDs in the non-clinical samples. These items can be incorporated into a future screening tool of BD for the general population.

### Ethics and consent to participate

This study was approved by the Institutional Review Board of the Samsung Medical Center (IRB Approval No. 2012-09-056). Written informed consent was obtained from all participants for the use of their test data in the BD studies of our research group.

### Consent to publish

Not applicable.

### Availability of data and materials

All the data supporting our findings is contained within the manuscript.

## Abbreviations

ANOVA: the analysis of variance
AUC: area under the curve
BD: bipolar disorders
BD1: bipolar I disorder
BD2: bipolar II disorder
DSM-IV: diagnostic and statistical manual of mental disorders, 4th edition
FDR: false discovery rate
HCL-32: hypomania Checklist 32
MDQ: mood disorder questionnaire
NPV: negative predictive value
PPV: positive predictive value
ROC: receiver operating characteristic
